# Hepatoprotective Role of *Hydrangea macrophylla* against Sodium Arsenite-Induced Mitochondrial-Dependent Oxidative Stress via the Inhibition of MAPK/Caspase-3 Pathways

**DOI:** 10.3390/ijms18071482

**Published:** 2017-07-10

**Authors:** Md Rashedunnabi Akanda, Hyun-Jin Tae, In-Shik Kim, Dongchoon Ahn, Weishun Tian, Anowarul Islam, Hyeon-Hwa Nam, Byung-Kil Choo, Byung-Yong Park

**Affiliations:** 1 College of Veterinary Medicine and Biosafety Research Institute, Chonbuk National University, Iksan 54596, Korea; rashed.mvd@gmail.com (M.R.A.); hjtae@jbnu.ac.kr (H.-J.T.); iskim@jbnu.ac.kr (I.-S.K.); ahndc@jbnu.ac.kr (D.A.); tianws0502@126.com (W.T.); anowarul.vet@gmail.com (A.I.); 2 Department of Pharmacology and Toxicology, Sylhet Agricultural University, Sylhet 3100, Bangladesh; 3 Department of Crop Science and Biotechnology, Chonbuk National University, Jeonju 54896, Korea; hh_hh@jbnu.ac.kr (H.-H.N.); bkchoo@jbnu.ac.kr (B.-K.C.)

**Keywords:** hepatoprotection, *Hydrangea macrophylla*, NaAsO_2_, mitogen-activated protein kinase (MAPK), caspase-3

## Abstract

Sodium arsenite (NaAsO_2_) has been recognized as a worldwide health concern. *Hydrangea macrophylla* (HM) is used as traditional Chinese medicine possessing antioxidant activities. The study was performed to investigate the therapeutic role and underlying molecular mechanism of HM on NaAsO_2_-induced toxicity in human liver cancer (HepG2) cells and liver in mice. The hepatoprotective role of HM in HepG2 cells was assessed by using 3-(4,5-dimethylthiazol-2-Yl)-2,5-diphenyltetrazolium bromide (MTT), reactive oxygen species (ROS), and lactate dehydrogenase (LDH) assays. Histopathology, lipid peroxidation, serum biochemistry, quantitative real-time polymerase chain reaction (qPCR) and Western blot analyses were performed to determine the protective role of HM against NaAsO_2_ intoxication in liver tissue. In this study, we found that co-treatment with HM significantly attenuated the NaAsO_2_-induced cell viability loss, intracellular ROS, and LDH release in HepG2 cells in a dose-dependent manner. Hepatic histopathology, lipid peroxidation, and the serum biochemical parameters alanine aminotransferase (ALT) and aspartate aminotransferase (AST) were notably improved by HM. HM effectively downregulated the both gene and protein expression level of the mitogen-activated protein kinase (MAPK) cascade. Moreover, HM well-regulated the Bcl-2-associated X protein (Bax)/B-cell lymphoma-2 (Bcl-2) ratio, remarkably suppressed the release of cytochrome *c*, and blocked the expression of the post-apoptotic transcription factor caspase-3. Therefore, our study provides new insights into the hepatoprotective role of HM through its reduction in apoptosis, which likely involves in the modulation of MAPK/caspase-3 signaling pathways.

## 1. Introduction

Inorganic arsenic compounds are heavy metal toxicants recognized as human carcinogens [1,2]. Among them, sodium arsenite is the most hazardous inorganic arsenic compound for human and animal health [3]. Arsenic is found in the environment surrounding the industrial and natural sources, raising eco-friendly public health concerns due to modern globalization [4]. Trivalent arsenical (arsenite) in ground water is the foremost source of arsenicosis, affecting more than 140 million people globally, particularly in India, Bangladesh, and neighboring countries [5]. Epidemiological investigations reveal links between arsenicosis and pathogenesis of various adverse health effects such as liver disorders, vascular diseases, diabetes, and cancer [6,7]. Studies at the molecular and cellular level show that arsenicosis enhances the production of reactive oxygen species (ROS) that causes DNA methylation, lipid peroxidation, increase oxidation of protein and disrupt enzymes [8,9]. Among them, oxidative stress due to the excessive release of free radicals has been implicated in NaAsO_2_-mediated damage in liver, kidney, heart, brain, skin and other tissues [10].

Oxidative stress is a fairly new but widely standard theory of NaAsO_2_-induced hepatotoxicity [11]. The increasing oxidative stress and thereby reduction of the endogenous antioxidant system during arsenic intoxication assists as a crucial factor in liver disorders. Particularly, it may lead to the several pathological conditions such as hepatic degeneration, alteration of lipid status and progressive fibrosis [12]. Generation of intracellular ROS leads to disruption in the matrix metalloproteinase (MMP), lipid peroxidation, carbonylation of protein, an imbalance in the Bcl-2-associated X protein (Bax)/B-cell lymphoma-2 (Bcl-2) ratio, and releases of cytochrome *c* following stimulation of the mitogen-activated protein kinase (MAPK) cascade [13]. Arsenic-mediated oxidative stress stimulates the MAPK cascade and induces apoptosis in hepatocytes via the mitochondria-dependent caspase signaling pathway [14,15].

Antioxidants have been recognized as favorable for mitigating arsenic-mediated oxidative stress in liver [16]. Naturally-found phytomedicine and their active ingredients have received significant attention as antioxidant agents and might offer some protection against oxidative stress, thus having a potential role in reducing the toxicity of trivalent arsenite [17]. Traditional herbal phytomedicine has received much attention as effective and alternative remedies for liver diseases [18]. In this study, we emphasize for the first time a simple and competent process of obtaining an extract from *Hydrangea macrophylla* (HM) seeds that have strong efficacy on NaAsO_2_-induced hepatotoxicity in vitro and in vivo. HM is a Hydrangeaceae plant native to the Korean mountains known as “soogook”, and is traditionally used as a folk medicine to treat many diseases such as diabetes and liver disorders. The major components of the HM extract such as phyllodulcin, hydrangenol, and hydrangeic acid has been determined by the high-performance liquid chromatography method [19]. The biological properties of HM and its active compounds have been reported with respect to antioxidant [20] anti-diabetic [21] and anti-malarial [22] activities. However, to our knowledge, HM has not been previously reported for its hepatoprotective effect. Therefore, based on the traditional uses and pharmacological actions of the active component of HM, our study investigates the hepatoprotective activities of HM and underlying molecular mechanisms involved in the action of NaAsO_2_-induced oxidative stress in liver.

## 2. Results

### 2.1. Analysis of Total Phenolic and Flavonoid Content of HM

Phenolic and flavonoid contents are the secondary metabolites of the plant, which exhibits a series of biological activities, and certainly, has antioxidant properties. The total phenolic and flavonoid contents of HM were investigated and are presented in Table 1.

### 2.2. HM Reduced NaAsO_2_-Induced Oxidative Stress in Human Liver Cancer (HepG2) Cells

**Figure 1:**
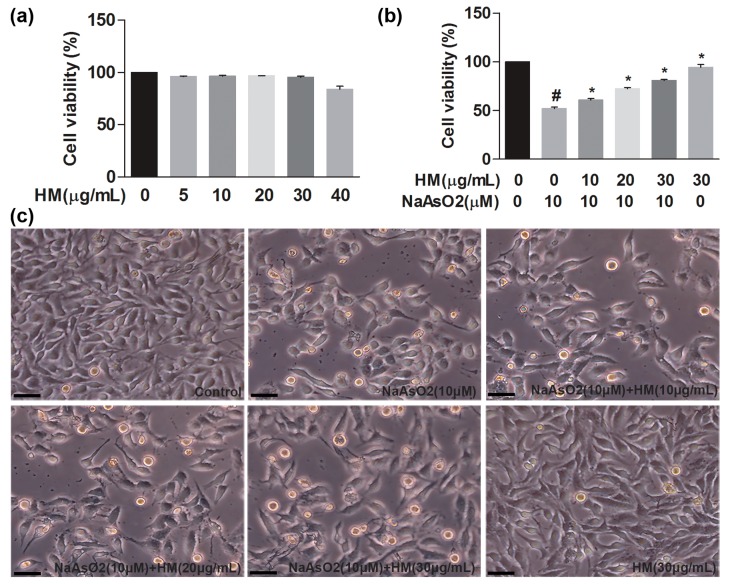
Hepatoprotective effects of HM against NaAsO_2_-induced oxidative stress in human liver cancer (HepG2) cells. (**a**) Cytotoxicity and (**b**) cell viability were measured by the 3-(4,5-dimethylthiazol-2-Yl)-2,5-diphenyltetrazolium bromide (MTT) assay; (**c**) observation of HepG2 cell morphology. Cells were pretreated with different concentrations of HM for 1 h, followed by co-treatment with NaAsO_2_ for 24 h. Scale bar: 200 μM. Data are expressed as mean ± standard error mean (SEM) of three independent experiments. # *p* < 0.05 compared with the control and NaAsO_2_ group, and * *p* < 0.05 compared with the NaAsO_2_ and HM extract-treated groups.

We performed the 3-(4,5-dimethylthiazol-2-Yl)-2,5-diphenyltetrazolium bromide (MTT) assay to evaluate the hepatoprotective effects of HM against NaAsO_2_-induced cytotoxicity in HepG2 cells. We first determined the optimum concentration of the HM extract and found that cell viability was more than 90% after 24 h of incubation with various concentrations of HM (5, 10, 20, and 30 μg/mL) (Figure 1a). After that, NaAsO_2_ markedly (*p* < 0.05) decreased cell viability as compared to the control. We found that HM (10, 20 and 30 μg/mL) significantly (*p* < 0.05) protected HepG2 cells against NaAsO_2_-mediated oxidative damage in a dose-dependent manner (Figure 1b). Likewise, the morphology of HepG2 cells was improved by co-treatment of the HM (Figure 1c). Additionally, HM (30 μg/mL) did not show any detrimental effect on HepG2 cells viability and morphology.

### 2.3. HM Decreased the Intracellular ROS Generation

**Figure 2:**
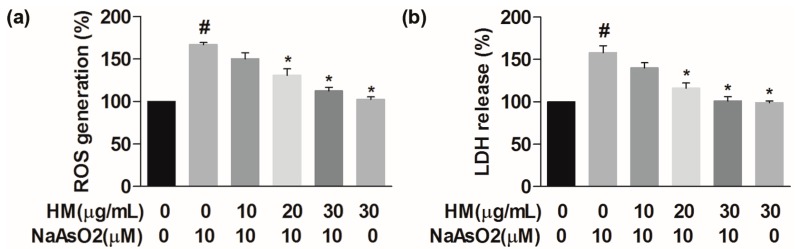
HM inhibited the reactive oxygen species (ROS) and lactate dehydrogenase (LDH) release in HepG2 cells. (**a**) Intracellular ROS and (**b**) LDH levels were measured. Cells were pretreated with different concentrations of HM (10, 20, and 30 μg/mL) for 1 h, followed by co-treatment with 10 µM NaAsO_2_ for another 24 h. Data are expressed as mean ± standard error mean (SEM) of three independent experiments. # *p* < 0.05 compared with the control and NaAsO_2_ group, and * *p* < 0.05 compared with the NaAsO_2_ and HM extract-treated groups.

Intracellular ROS generation followed by accumulation of free radicals is supposed to be an important marker for understanding NaAsO_2_-induced hepatic cell death. To investigate the role of HM on NaAsO_2_-induced ROS generation, HepG2 cells were pretreated with HM (10, 20, and 30 μg/mL) for 1 h and subsequently exposed to HM and NaAsO_2_ (10 μM) for another 24 h. NaAsO_2_ markedly (*p* < 0.05) increased the generation of intercellular ROS as compared to the control. Conversely, co-treatment with the HM (20 and 30 μg/mL) significantly (*p* < 0.05) and dose-dependently reduced ROS generation (Figure 2a). Also, HM (30 μg/mL) did not show any effects on the intracellular ROS generation. These data showed that HM protects hepatic cells from the oxidative damaging effect caused by NaAsO_2_. 

### 2.4. HM Inhibited the Lactate Dehydrogenase (LDH) Release

In vitro hepatoprotective effect of HM was determined by performing LDH assay using HepG2 cells culture supernatant. The LDH level was significantly (*p* < 0.05) increased in NaAsO_2_ exposed cells compared to the control; however, co-treatment with the HM (20 and 30 μg/mL) significantly (*p* < 0.05) and dose-dependently reduced the LDH release (Figure 2b). Besides, HM (30 μg/mL) alone did not show any effect on the LDH release. Our data revealed that HM protects the HepG2 cells from cytotoxicity caused by NaAsO_2_.

### 2.5. HM Improved the Liver Histopathology and Body Weight

**Figure 3:**
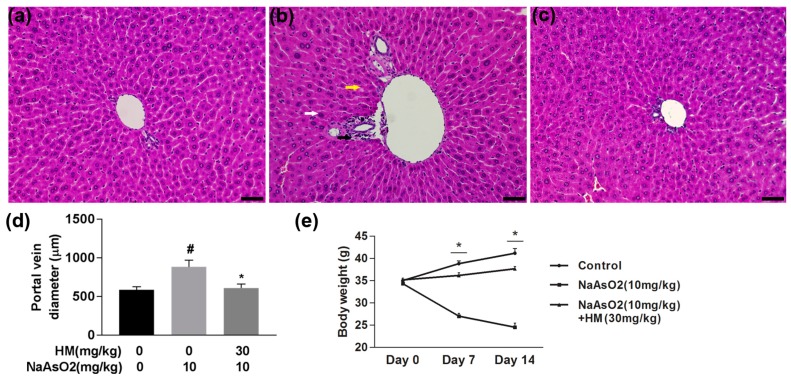
HM improved the liver histology and body weight in experimental mice. Untreated mice were used as a control to compare histological changes induced by NaAsO_2_. (**a**) Normal control; (**b**) NaAsO_2_ (10 mg/kg); (**c**) Co-treatment with HM (30 mg/kg) + NaAsO_2_ (10 mg/kg); (**d**) portal vein diameter; and (**e**) Body weight. In the NaAsO_2_ group, the white arrow indicates the degenerative nucleus, the yellow arrow indicates the blurred cytoplasm and the black arrow indicates the damaged hepatic lobule surrounding hepatic artery. In contrast, co-treatment with HM improved histological changes compared to NaAsO_2_ alone. Portal vein diameter and body weight also significantly decreases and increased in HM-treated mice, respectively. Data are expressed as mean ± standard error mean (SEM) of three independent experiments. Scale bar: 200 μM. # *p* < 0.05 compared with the control and NaAsO_2_ group, and * *p* < 0.05 compared with the NaAsO_2_ and HM extract-treated group.

NaAsO_2_-mediated liver damage and its protection by HM treatment in mice were confirmed by microscopic evaluation of histopathological changes. Microscopic analysis of the liver indicated a normal structure of hepatocytes arranged around the portal vein in the control mice liver (Figure 3a); however, the NaAsO_2_-exposed group showed damage in the hepatic lobules surrounding the hepatic artery, degenerated nuclei, a dilated portal vein, and blurred cytoplasm (Figure 3b). The histopathological changes induced by NaAsO_2_ were considerably improved by co-treatment with the HM (Figure 3c). The dilated portal vein diameter was also markedly (*p* < 0.05) reduced by HM co-treatment (Figure 3d). Moreover, arsenic-exposed mice showed a significant (*p* < 0.05) decline in body weight compared to normal control mice, whereas co-administration of HM and NaAsO_2_ effectively (*p* < 0.05) increased body weight compared to treatment with NaAsO_2_ alone (Figure 3e).

### 2.6. HM Regulated the Serum Biochemical Parameters

**Figure 4:**
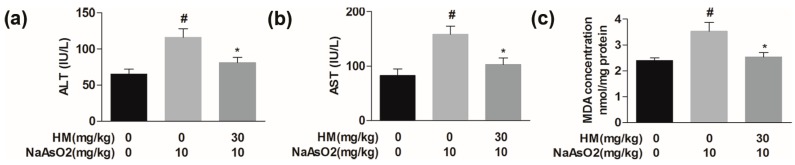
Effect of HM on serum markers and lipid peroxidation in experimental mice. (**a**) Serum alanine aminotransferase (ALT); (**b**) serum aspartate aminotransferase (AST), and (**c**) Malondialdehyde (MDA) levels in liver tissue. ALT, AST and MDA levels were increased in NaAsO_2_-intoxicated mice. Co-treatment with HM significantly decreased the serum ALT and AST and tissue MDA levels as compared to NaAsO_2_ alone. Data are expressed as mean ± standard error mean (SEM) of three independent experiments. # *p* < 0.05 compared with the control and NaAsO_2_ group, and * *p* < 0.05 compared with the NaAsO_2_ and HM extract-treated group.

Alanine aminotransferase (ALT) and aspartate aminotransferase (AST) levels were investigated as biomarkers of liver cell integrity. The levels of the serum cytosolic enzymes ALT and AST were considerably (*p* < 0.05) higher in NaAsO_2_-intoxicated mice than in normal control. We found that co-treatment with HM significantly (*p* < 0.05) improved liver physiology by reducing the level of ALT and AST as compared to NaAsO_2_ alone (Figure 4a,b). These data indicate that HM improves the liver physiology in NaAsO_2-_mediated hepatotoxicity.

### 2.7. HM Controlled the Lipid Peroxidation Production

Thiobarbituric acid reactive substances (TBARS) concentration was evaluated to determine the level of malondialdehyde (MDA) in liver samples, which is the end-product of lipid peroxidation in oxidative stress. The MDA level in the NaAsO_2_ group was significantly (*p* < 0.05) higher than in the control. The elevated level of MDA was markedly (*p* < 0.05) reduced after co-treatment with the HM (Figure 4c). This result suggested that HM might have anti-oxidative effect against NaAsO_2_-induced hepatic damage in mice. 

### 2.8. HM Suppressed the Gene Expression of MAPKs (Extracellular Signal-Regulated Kinases (ERK), C-Jun N-Terminal Kinases (JNK), and p38)

**Figure 5:**
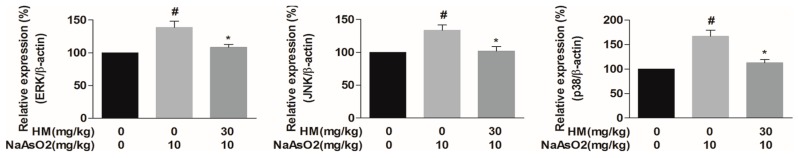
HM attenuated the gene expression of mitogen-activated protein kinase (MAPK) (extracellular signal-regulated kinases (*ERK*), C-Jun N-terminal kinases (*JNK*), and *p38*) in liver tissue. The expression level of *MAPK* genes was significantly upregulated in NaAsO_2_-exposed liver tissue, but co-treatment with HM effectively downregulated the gene expression. Data are expressed as mean ± standard error mean (SEM) of three independent experiments. # *p* < 0.05 compared with the control and NaAsO_2_ group, and * *p* < 0.05 compared with the NaAsO_2_ and HM extract-treated group.

To reveal the possible molecular pathways of hepatoprotection by HM, we evaluated gene expression in liver homogenates by quantitative real-time polymerase chain reaction (qPCR) analysis. Treatment with NaAsO_2_ markedly (*p* < 0.05) increased the gene expression level of *ERK*, *JNK*, and *p38* compared to the control, and these higher gene expression level were significantly (*p* < 0.05) attenuated by co-treatment with HM compared with NaAsO_2_ alone (Figure 5). These effects suggest that HM significantly inhibits the gene expression of MAPKs, thereby reducing liver damage caused by NaAsO_2_.

### 2.9. HM Mitigated NaAsO_2_-Mediated Hepatotoxicity by Regulating Anti-Apoptotic Signaling Pathways 

**Figure 6:**
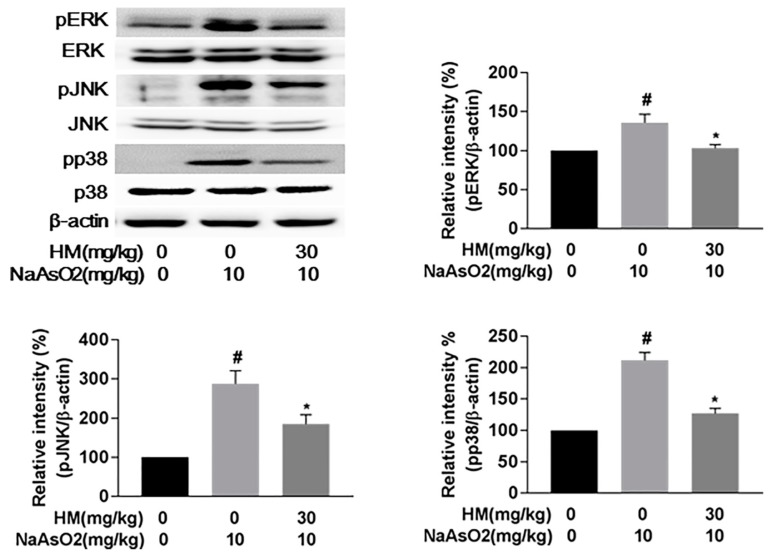
Effect of HM on the MAPK (pERK1/2, pJNK, and pp38) signaling pathway in liver tissue. The expression of pERK, pJNK, and pp38 in liver tissue was analyzed by Western blot. Co-treatment with HM significantly downregulated the expression level of pERK1/2, pJNK, and pp38. Data are expressed as mean ± standard error mean (SEM) of three independent experiments. # *p* < 0.05 compared with the control and NaAsO_2_ group, and * *p* < 0.05 compared with the NaAsO_2_- and HM extract-treated group.

**Figure 7:**
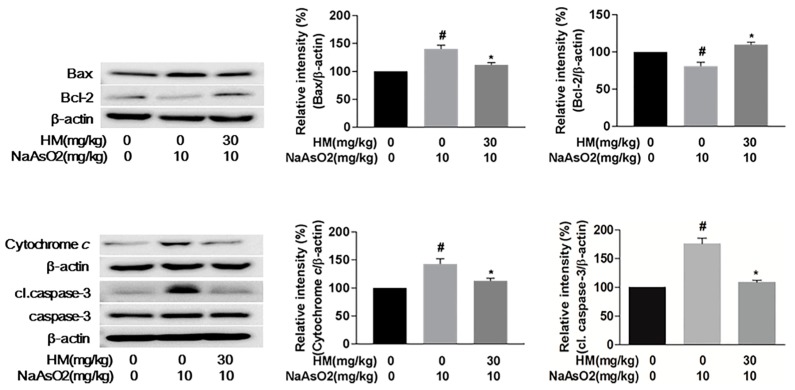
Effect of HM on the apoptotic signaling pathways in response to NaAsO_2_ and HM exposure. The expression of Bcl-2-associated X protein (Bax), B-cell lymphoma-2 (Bcl-2), cytochrome *c*, and caspase-3 in liver tissue was analyzed by Western blot analysis. Co-treatment with HM effectively regulated the Bax/Bcl-2 ratio and notably downregulated the expression of cytochrome *c*, and cleaved caspase-3. Data are expressed as mean ± standard error mean (SEM) of three independent experiments. # *p* < 0.05 compared with the control and NaAsO_2_ group, and * *p* < 0.05 compared with the NaAsO_2_ and HM extract-treated group.

Mitochondrial damage is an important marker of apoptotic cell death and is executed through ROS-mediated oxidative stress [23]. Here, we evaluated the involvement of the mitochondrial pathway of hepatic apoptosis by western blot analysis. Our results revealed that NaAsO_2_ markedly (*p* < 0.05) upregulated phosphorylation of the MAPK cascade (pERK1/2, pJNK, and pp38), whereas co-treatment with HM effectively (*p* < 0.05) downregulated the phosphorylation level (Figure 6). The expression ratio of the Bax/Bcl-2 family protein was also significantly (*p* < 0.05) controlled by HM co-treatment. Meanwhile, the expression of cytochrome *c* and activated caspase-3 was also remarkably (*p* < 0.05) increased with NaAsO_2_ treatment. However, HM co-treatment significantly (*p* < 0.05) attenuated the cytochrome *c* and activated caspase-3 expression (Figure 7). Together, these results supported that HM considerably regulates the mitochondrial-dependent hepatic damage caused by NaAsO_2_.

## 3. Discussion

Sodium arsenite (NaAsO_2_) is a ubiquitous environmental stressor that has become a danger to human and animal health [24]. Long-term exposure to arsenic compounds has been directly related to major health disorders such as hepatitis, hepatic cancer, diabetes, coronary disease, stroke, peripheral vascular disease, and skin disease [25,26]. Among them, the liver is the most target site for arsenic toxicity due to its physiology, particularly for biochemical alteration of arsenic metabolites. Oxidative injury plays a vital role in such kinds of alteration-related pathophysiology. Treatment preventing the hepatic damage may lead to prospective therapeutic strategies against the hepatic disorders and HM extract may provide a novel therapeutic candidate. Evidence has stated that extract from HM has potential antioxidative properties [21]. In this study, we demonstrated that HM can be used as a novel indigenous phytomedicine due to its strong hepatoprotective effects against NaAsO_2_-mediated oxidative stress in vitro and in vivo.

Phenolics and flavonoids are the most important plant secondary metabolites and have the strong antioxidant capacity [27,28]. Their antioxidant ability is mainly due to their redox properties, which allow them to act as reducing agents, oxygen scavengers and transition metal ions chelator [29]. In our study, we found a considerable amount of phenolic and flavonoid content in HM extract that may be the major contributor for the antioxidant role against oxidative stress-induced hepatic damage. NaAsO_2_-mediated cytotoxicity is mainly associated with the generation of ROS, increase in lipid peroxidation, DNA dysfunction, cell cycle disruption, and apoptotic cell death [30,31]. An in vitro hepatocellular model, HepG2 cells were exposed to NaAsO_2_ and led to cell death by oxidative stress. However, with pretreatment with HM, the cell viability was restored, indicating its hepatoprotective role. Intracellular ROS release and LDH production have established a mechanism that is associated with hepatic cell death [5]. Excessive accumulation of intracellular ROS and LDH production may accelerate unstable cellular homeostasis that leads to mitochondrial membrane dysfunction [32]. Natural phytomedicine, with the capability for scavenging free radicals, may reduce conditions correlated to oxidative stress. We found that exposure to NaAsO_2_ in HepG2 cells considerably increased the ROS and LDH release that boosted the oxidative stress and prompted cell apoptosis. However, dose-dependent and significant inhibition of ROS generation and LDH leakage in HepG2 cells were observed after HM treatment. Therefore, HM may protect the cells against NaAsO_2_-induced oxidative stress via its antioxidant capacity.

To evaluate either HM exhibits the same defensive role in vivo, NaAsO_2_-intoxicated mice were studied. In an attempt to assess the internal hepatotoxicity by NaAsO_2_, the histopathological changes were evaluated. We observed that arsenic caused hepatic tissue destruction, degenerated the nucleus, dilated the portal vein, and blurred cytoplasm, perhaps due to the accumulation of free radicals and following lipid peroxidation. Such findings are related to the previous study [33]. Co-treatment with HM during arsenic exposure effectively improved the hepatic histological architecture. The serum biochemical indicators ALT and AST were positively correlated with hepatic histopathology [34]. The activities of ALT and AST are frequently used as a diagnostic marker of liver damage since they are linked with liver physiology [35]. Increased levels of ALT and AST in the bloodstream damage the hepatic cell integrity. We found HM was effective in restoring the serum enzyme biomarkers. MDA is the end product of lipid peroxidation and is a well-established and standard mechanism of cellular injury used as an indicator of oxidative stress in cells and tissues [36]. We found the significant increase of MDA level in arsenic-exposed liver suggested higher lipid peroxidation, leading to tissue damage. However, HM treatment effectively attenuated the MDA that plays an important role in inhibiting oxidative stress [37]. At the end of the experiment, a significant increase in body weight was recorded in HM-treated mice compared to the NaAsO_2_ group alone.

Multiple biological mechanisms and molecular signaling pathways are involved in apoptotic cell death [38]. Mitochondria play a crucial role in the induction of cellular apoptosis [39]. Cytotoxic ROS activate mitochondrial-dependent apoptosis via stimulation of the MAPK cascade and subsequent modulation of the pro-apoptotic protein Bax and anti-apoptotic protein Bcl-2, followed by cytochrome *c* release, and finally executes hepatic cell death by activation of the caspase cascade pathway [25,40]. The relevant observation was confirmed in this study. We observed that NaAsO_2_ markedly increased the gene expression and phosphorylation of the MAPK cascade that ultimately leads to apoptosis, while HM effectively reduced both the gene expression and phosphorylation of the MAPK cascade in arsenic-exposed liver tissue. Likewise, HM notably decreased the expression of Bax and increased expression of the Bcl-2 protein, subsequently controlling the release of cytosolic cytochrome *c*. These findings suggest that HM positively regulates the NaAsO_2_-induced mitochondrial-dependent apoptotic signaling pathway. It is well established that Bax/Bcl-2 proteins control permeabilization of the mitochondrial membrane and thus control the release of apoptotic factors from the intermembrane space of mitochondria [25].

An imbalance in the Bax/Bcl-2 ratio leads to the production of cytochrome *c* from mitochondria and subsequently activates the apoptotic protein caspase to trigger cellular apoptosis [41,42]. Cytochrome *c* promotes the ATP-dependent formation of the apoptosome, resulting in activation of the caspase cascade via a mitochondrial-dependent pathway [43]. Predominantly, overactivation of caspase-3 indicates pathogenesis in hepatic cell death [5,44]. We found that NaAsO_2_ markedly upregulated the expression of caspase-3, whereas notable attenuation of caspase-3 was found after co-treatment with HM. This result was supported by earlier investigation [45]. Therefore, our data revealed that HM could protect the liver from NaAsO_2_-induced oxidative stress through the mitochondria-dependent pathway, indicating that the radical scavenging potency of HM is responsible for its anti-apoptotic activity.

## 4. Materials and Methods

### 4.1. Chemicals and Antibodies

The highest analytical grades of all chemicals were used. Sodium arsenite (NaAsO_2_), 3-(4,5-dimethylthiazol-2-Yl)-2,5-diphenyltetrazolium bromide (MTT), penicillin/streptomycin, gallic acid, rutin, hematoxylin, eosin, and protease inhibitor were purchased from Sigma-Aldrich (St. Louis, MO, USA). Fetal bovine serum (FBS), Dulbecco’s Modified Eagle’s Medium (DMEM), and other cell culture reagents were obtained from Gibco (Carlsbad, CA, USA). Dimethyl sulfoxide (DMSO) was obtained from Bioshop (Burlington, ON, Canada). RNA extraction kits were purchased from RiboEx and Hybrid-R (Gene All, Seoul, South Korea). Tissue protein extraction reagent (T-PER), complementary DNA (cDNA) synthesis (ReverTra Ace^®^ qPCR RT Kit, Toyobo, Osaka, Japan), and bicinchoninic acid (BCA) protein assay kits were purchased from Thermo Scientific (Waltham, MA, USA). The SYBR Green qPCR kit was purchased from Toyobo (Osaka, Japan). Primary antibodies (pERK1/2, pJNK, pp38, tERK1/2, tJNK, tp38, Bax, Bcl-2, cytochrome *c*, cleaved caspase-3, and caspase-3) and β-actin were purchased from Cell Signaling (Danvers, MA, USA). The goat anti-rabbit immunoglobulin G horseradish peroxidase (IgG-HRP) secondary antibody was purchased from Santa Cruz (Santa Cruz, CA, USA). The WESTSAVE Gold Enhanced Chemiluminescence (ECL) detection kit was acquired from Abfrontire (Seoul, Korea), and the ALT and AST kits were from ASAN (Hwaseong, Korea). LDH cytotoxicity assay kit was obtained from TAKARA (Tokyo, Japan), and the ROS-Glo H_2_O_2_ assay kit was from Promega (Madison, WI, USA). Zoletil 50 was supplied by Virbac S.A (Carros, France). 

### 4.2. Preparation of Hydrangea Macrophylla Seed Extract

The seeds of *Hydrangea macrophylla* (HM) plant were collected from Jirisan located in the southern part of South Korea and authenticated based on its microscopic and macroscopic features by the Korea Institute of Oriental Medicine. We prepared HM seeds extract according to the previously described method with minor modifications [46]. Briefly, the plant seeds were sliced and dried completely. The extract was prepared by maceration of seeds sample with 70% ethanol (twice for 2 h reflux), and then filtered extract was concentrated under vacuum centrifuge and dehydrated with a lyophilizer. The powder extract was liquefied in DMSO and sterilized using a 0.22-μm syringe filter. The dried extract was kept at −20 °C. The study was conducted using a single batch of plant extract to avoid batch-to-batch variation and maximize the product constancy.

### 4.3. Determination of Total Phenolic and Flavonoid Content

Total phenolic and flavonoid content of HM extract was measured according to the previously described method [47].

### 4.4. Cell Culture

HepG2 cells were maintained at 37 °C in a 5% CO_2_ humidified incubator. Cells were cultured in DMEM supplemented with 10% FBS and 1% penicillin and streptomycin. The cell culture medium was changed for every 2 days, and the cells were subcultured when they reached about 90% confluency in the culture flask.

### 4.5. Assessment of Cell Viability 

MTT assay was used to measure cell viability. HepG2 cells were seeded (1 × 10^4^ cells/well in 96-well plates) and cultured in a 37 °C incubator overnight. For evaluating the cytotoxicity of HM, cells were treated with HM (5, 10, 20, 30 and 40 μg/mL) for 24 h. In contrast, measuring the cell viability, cells were pretreated for 1 h with different concentrations of HM (10, 20 and 30 μg/mL) and then co-incubated with HM and NaAsO_2_ (10 μM) for an additional 24 h. The medium was replaced with 0.5 mg/mL of the MTT working solution and incubated for 2 h. The blue formazan crystals were solubilized by DMSO. Optical density was measured at 570 nm absorbance by a tunable versa max microplate reader (Molecular Devices, Sunnyvale, CA, USA). Similarly, for observation of HepG2 cell morphology, the image of the cell was captured by an inverted microscope (Olympus, CKX41, Tokyo, Japan) at fixed 100× magnification. 

### 4.6. Measurement of Reactive Oxygen Species (ROS) Generation

To evaluate the level of intracellular ROS, HepG2 cells (1 × 10^4^ cells/well) were cultured in 96-well plates overnight. After adherence, cells were pretreated for 1 h with different concentrations of HM (10, 20 and 30 μg/mL) and then co-incubated with HM and NaAsO_2_ (10 μM) for an additional 24 h. The intracellular ROS level was measured according to the manufacturers’ procedure for the kit, and the absorbance was measured at 490 nm using a tunable versa max microplate reader.

### 4.7. Determination of Lactate Dehydrogenase (LDH) Release

To measure the level of extracellular LDH release, HepG2 cells (1 × 10^4^ cells/well) were cultured in 96-well plates overnight. After adherence, cells were pretreated for 1 h with different concentrations of HM (10, 20 and 30 μg/mL) and then co-incubated with HM and NaAsO_2_ (10 μM) for an additional 24 h. The LDH level was measured according to the manufacturers’ procedure of kit, and the absorbance was measured at 490 nm using a tunable versa max microplate reader.

### 4.8. Mice Management and Experimental Design

Male ICR mice (6 weeks old) were maintained in accordance with the animal welfare regulations of the Institutional Animal Care and Use Committee (IACUC; CBNU 2016-68), Chonbuk National University Laboratory Animal Centre, South Korea. Mice were kept in standard mouse cages with an ad libitum supply of food and distilled water. Ideal conditions for temperature (23 ± 2 °C), humidity (35–60%), and photoperiod cycle (12 h light and 12 h dark) were maintained over the experimental period. Mice were adapted to the laboratory conditions for 1 week before starting the experiment. A total of 36 mice were randomly divided into three groups: (1) normal control mice were treated with saline; (2) toxic control mice were treated with NaAsO_2_ once daily (10 mg/kg body weight, (per os/orally) p.o., for 10 days); and (3) experimental mice were treated with HM once daily (30 mg/kg body weight, p.o., for 15 days) prior to treatment with NaAsO_2_ (10 mg/kg body weight, p.o., for 10 days). After the experimental period, mice were fasted overnight and anesthetized with Zoletil 50.

### 4.9. Histopathological Study of the Liver

Mice liver was collected for histopathological examination. Liver samples were immediately fixed in 10% neutral buffered formalin (NBF) and processed in an auto processor (Excelsior ES, Thermo Scientific, Waltham, MA, USA). After embedding in paraffin, 5-μm sections were stained with hematoxylin and eosin and mounted on glass slides. Digital images were obtained using a Leica DM2500 microscope (Leica Microsystems, Wetzlar, Germany) at a fixed 200× magnification. The diameter of the portal vein was measured using image measurement software (v 22.1., iSolution DTM, Vancouver, BC, Canada). 

### 4.10. Serum Biochemical Analysis

The levels of the basic liver function biomarker enzymes serum ALT and AST was examined. Blood samples were collected from the mouse and incubated for 30 min at room temperature. Blood was centrifuged at 3000 rpm for 15 min at 4 °C to collect the serum. ALT and AST levels were analyzed according to the manufacturer’s recommendation. 

### 4.11. Lipid Peroxidation Assay

The level of MDA is an important marker of oxidative stress condition. MDA concentration was measured in the liver tissue. The samples homogenized in a ratio of 1/10 in 1.15% (*w*/*v*) ice-cold KCl solution with the aid of the thiobarbituric acid (TBA) established method [48]. The standards of 2.5, 5, 10 and 20 nmol/mL tetra ethoxy propane (TEP) were used. The results were expressed as nmol MDA/mg protein.

### 4.12. Quantitative Real-Time Polymerase Chain Reaction (qPCR) Analysis

Total RNA was isolated from mouse liver tissue according to the manufacturer’s instructions, and RNA concentration was quantified using a BioSpec-nano spectrophotometer (Shimadzu Biotech, Tokyo, Japan) at a 260/280 nm ratio. For cDNA synthesis, 3 μg of total RNA was used, and the cDNA synthesis procedure was performed according to the manufacturer’s instructions. qPCR was performed using the SYBR Green Real-Time PCR master mix with the Roche LightCyclerTM, and the conditions were maintained according to the manufacturer’s instructions. β-Actin was used as the housekeeping gene. Relative expression of target genes was normalized with reference gene (β-actin). The nucleotide sequences of the primers are presented in Table 2 [49].

### 4.13. Western Blot Analysis

Liver lysates were prepared in ice-cold lysis buffer containing tissue protein extraction reagent (T-PER), phenylmethanesulfonyl (PMSF), Na_3_VO_4_ (sodium orthovanadate), and protease inhibitor cocktail. The lysate was centrifuged at 12,000 rpm for 20 min at 4 °C, and the supernatant was collected. The total protein concentration of the lysate supernatant was measured using the BCA protein assay kit. An equal amount of protein was separated by 12% sodium dodecyl sulfate-polyacrylamide gel electrophoresis (SDS-PAGE) and transferred to a nitrocellulose membrane. The membrane was blocked with a regular blocking solution (5% non-fat skim milk in Tris-buffered saline (TBST)) for 1 h at room temperature, followed by incubation with primary antibodies against pERK, pJNK, pp38, Bax, Bcl-2, cytochrome *c*, caspase-3, cleaved caspase-3, and β-actin overnight at 4 °C. The blot was washed and then incubated with anti-rabbit secondary antibodies for 1 h at room temperature. Protein band was detected using an ECL detection kit, and images were obtained using an imaging system (LAS-400 image system, GE Healthcare, UK). β-actin was used as the control. 

### 4.14. Statistical Analysis

Data were analyzed with Graph Pad Prism 7.0 (La Jolla, CA, USA) and expressed as mean ± standard error mean (SEM). Group comparisons were performed using analysis of variance (ANOVA), followed by Tukey’s multiple comparisons tests. The minimum statistical significance was considered *p* < 0.05 for all analyses.

## 5. Conclusions

In conclusion, both in vitro and in vivo findings offer evidence of the hepatoprotective potency of HM on NaAsO_2_-mediated oxidative damage via attenuation of free radical generation, restoration of hepatic physiology, and reduction in mitochondrial-dependent apoptosis. Thus, HM is a natural phytomedicine that seems to be a promising therapeutic agent for treatment of hepatic disorders by targeting oxidative stress.

## Figures and Tables

**Table 1:** Total phenolics, flavonoids and extraction yield of *Hydrangea macrophylla* (HM).

Plant Extract	Total Phenolics (mg Gallic Acid Equivalent/g Extract)	Total Flavonoids (mg Rutin/g Extract)	Total Yield (%)
HM extract	92.358 ± 0.342	220.725 ± 3.263	26.9

**Table 2:** Nucleotide sequences of the primers for qPCR

Gene	Primers Sequence (5′–3′)	Size (bp)	Genebank Accession No.
*ERK*	TCAGAGGCAGGTGGATCTCT ACGGGGAGGACTCTGTTTTT	109	NM_011949.3
*JNK*	CGGAACACCTTGTCCTGAAT CACATCGGGGAACAGTTTCT	93	NM_016700.4
*p38*	AGCCAATTCCAGTGTTGGAC TTCTGGGCTCCAAATGATTC	120	NM_011951.3
*β-actin*	AGAAGATCTGGCACCACACC TACGACCAGAGGCATACAGG	195	NM_007393.5
